# Editorial: Integration of evidence-based research and practice in preventive and pediatric dentistry

**DOI:** 10.3389/froh.2022.1017226

**Published:** 2022-09-26

**Authors:** Jayakumar Jayaraman, Sreekanth Kumar Mallineni

**Affiliations:** ^1^ Department of Pediatric Dentistry, Virginia Commonwealth University School of Dentistry, Richmond, VA, United States; ^2^ Center for Transdisciplinary Research (CFTR), Saveetha Institute of Medical and Technical Sciences, Saveetha Dental College, Saveetha University, Chennai, Tamil Nadu, India; ^3^ Division for Globalization Initiative, Liaison Center for Innovative Dentistry Graduate School of Dentistry, Tohoku University, Sendai, Japan

**Keywords:** evidence based dentistry, pediatric dentistry, trends (source: meSH NLM), systematic review and meta-analysis, clinical practice guideline (CPG), randomized controled trials

**Editorial on the Research Topic** Integration of evidence-based research and practice in preventive and pediatric dentistry

Research in pediatric dentistry has been through an abstruse journey from its inception in the 1900s. Historically, scientific research in pediatric dentistry has shifted its focus from publishing case reports to research aimed at answering focused questions. The introduction of evidence-based research opened new directions and possibilities that provided solutions for addressing several clinical problems in pediatric dentistry. The foundation of evidence-based dentistry relies on the integration of the best scientific evidence, the expertise of the clinician, and most importantly, the treatment need and values of the patient.

## Evidence-based research

In evidence-based research, hierarchy is determined by the nature of the study design and the methodological quality of the study. This is portrayed in the form of a pyramid with weaker study designs at the bottom followed by case-control and cohort studies in the middle, randomized controlled trials (RCTs), and systematic reviews and meta-analysis at the very top. Although this hierarchy is based on the study design, the quality varies based on the internal and external validity of the individual study designs. Several tools and checklists have been developed to assess the quality of published studies. For example, Grading of Recommendations, Assessment, Development, and Evaluations (GRADE) provides a systematic approach to rating the certainty of evidence in systematic reviews (1). This considers the risk of bias, imprecision, inconsistency, indirectness, and publication bias of individual studies included in the analysis. Even in a well-conducted and reported systematic review, a “very low” certainty of the evidence of the included studies in which the true effect is probably markedly different from the estimated effect reduces the quality of evidence and thereby makes the weak recommendation for intervention.

## Current trends

Evidence-based research in Pediatric Dentistry mainly revolves around systematic reviews and meta-analysis that has gained popularity in the past few decades. More recently, newer dimensions towards synthesizing data within systematic reviews have been explored in Pediatric Dentistry including Network Meta-Analysis (NMA) and Trial Sequential Analysis (TSA). Historically, this approach has been employed in studies pertaining to clinical medicine. Network Meta-Analysis uses the estimates of the relative effectiveness of all interventions on the primary outcomes by combining direct and indirect evidence (2). Trial Sequential Analysis avoids random errors and calculates the required information size to detect or reject a certain intervention effect from the meta-analysis of primary outcomes (3).

A well-conducted systematic review of methodologically sound randomized controlled trials is mostly placed at the top of the evidence pyramid. Although this is the case, not all published systematic reviews are of good quality. There are two main aspects to determine the quality of a published study, firstly, to see how well it is conducted and secondly, how well it is reported. The guidelines on methodological quality inform authors on conducting research and it is recommended to follow them whilst designing the study. This will ensure adherence to the study design and identify any deviations from the initial study design. To assess the methodological quality of systematic reviews, A Measurement Tool to Assess Systematic Reviews (AMSTAR) has been developed (4). Using this tool, a recent study found that the reporting quality of systematic reviews and meta-analysis in Pediatric dentistry was inadequate and identified several areas for improvement (5). The same applied to the abstracts of systematic reviews and meta-analysis in Pediatric dentistry (6). In contrast to methodological quality, checklist items on the reporting quality provide authors a set of guidelines on transparent reporting and to avoid selective bias in reporting of results. For example, the Preferred Reporting Items for Systematic Reviews and Meta-Analyses (PRISMA) help authors to improve the reporting of systematic reviews (7). Most often, the methodological and reporting guidelines are related and go hand in hand. The reporting guidelines and the checklist items for other study designs are presented in the Enhancing the Quality and Transparency of Health Research (EQUATOR) network (www.equator-network.org). The above statements were developed specifically for different study designs and could be applied to any specialty in dentistry. Alternatively, a new set of evidence based recommendations for reporting research specific to Pediatric Dentistry has been recently presented by the Reporting Standards for Research in Pediatric Dentistry (RAPID) group. This statement was intended to facilitate complete and transparent reporting and thereby minimizing bias arising from inadequate reporting of research in Pediatric Dentistry (8).

Most recently, a very few umbrella reviews have been published on topics in Pediatric Dentistry (9, 10). Umbrella review is a review of previously published systematic reviews or meta-analysis and follows a uniform approach for all factors to allow their comparison. In simple terms, based on the design, an umbrella review is a systematic review of systematic reviews. To date, they represent one of the highest levels of evidence synthesis. Some key points to be considered towards conducting a robust umbrella review include specification of the protocol, the definition of the variables of interest, estimation of common effect size, reporting the heterogeneity and potential biases, performing stratification of the evidence, conducting sensitivity analyses, reporting transparent results, use of appropriate software and acknowledgment of the limitations (11). Another area of EBD is clinical practice guidelines (CPG) that are systematically developed statements to assist practitioner and patient decisions about specific clinical circumstances in Pediatric Dentistry. This is conducted by synthesizing the evidence from systematic reviews as well as evaluation of independent studies pertaining to a topic. The Academy of Pediatric Dentistry has published several such guidelines, for example, clinical practice guidelines on pulp therapy in primary teeth (12).

## Future directions

**Figure 1:**
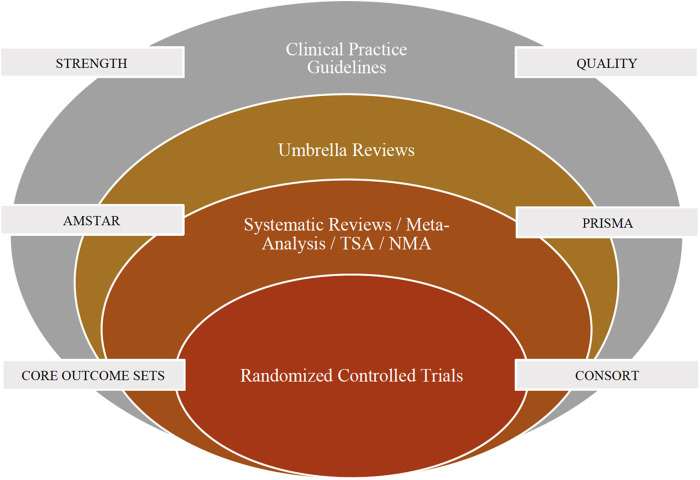
Integration of evidence based research specific to randomized controlled trials in pediatric dentistry. NMA, Network Meta-Analysis; TSA, Trial Sequential Analysis; CONSORT, Consolidated Standards of Reporting Trials; AMSTAR, A Measurement Tool to Assess Systematic Reviews; PRISMA, Preferred Reporting Items for Systematic Reviews and Meta-Analyses.

Any form of secondary research solely relies on the quality of primary research and the existing research materials. For example, a systematic review and meta-analysis (secondary research) on randomized trials (primary research) cannot provide a strong recommendation if the randomized trials were not conducted properly although the review strictly adhered to methodological (AMSTAR) and reporting (PRISMA) guidelines. It is important to improve the quality of primary research which will in turn lead to good quality secondary research. For example, there is no agreement amongst dental professionals or patients as to which outcomes should be measured when investigating interventions for clinical conditions related to pediatric dentistry. The Core Outcome Measures in Effectiveness Trials (COMET) is an initiative that aims at developing and applying agreed standardized sets of outcomes, known as “core outcome sets” (COS) that represents the minimum criteria that should be measured and reported in all clinical trials, for a specific condition (13). For clinical trials in pediatric dentistry, it is recommended to develop core outcome sets for various clinical scenarios. Also, it is recommended to follow the Consolidated Statement of Reporting Trials (CONSORT) guidelines whilst reporting trials in Pediatric Dentistry (14). The integration of evidence-based research specific to Randomized Controlled Trials in Pediatric Dentistry is presented in Figure 1.

In conclusion, future research on EBD in Pediatric Dentistry should focus on identifying deficiencies in both primary and secondary research that will in turn help enable providing scientifically valid recommendations. Consensus should be reached amongst the authors, editors, and other stakeholders on strictly adhering to the methodological and reporting guidelines or their extension when available. It is welcoming news that several pediatric dental journals have made already it compulsory for the authors to submit the reporting checklist whilst submitting their manuscripts. Researchers in clinical medicine has always been a forerunner, and it is time for the scientific community to adapt to the recent advancement in the synthesis and presentation of primary data in Pediatric Dentistry.

## References

[1] Guyatt GH Oxman AD Vist GE Kunz R Falck-Ytter Y Alonso-Coello P GRADE: an emerging consensus on rating quality of evidence and strength of recommendations. Br Med J. (2008) 336(7650):924–6. 10.1136/bmj.39489.470347.AD 18436948 PMC2335261

[2] Urquhart O Tampi MP Pilcher L Slayton RL Araujo MWB Fontana M Nonrestorative treatments for caries: systematic review and network meta-analysis. J Dent Res. (2019) 98(1):14–26. 10.1177/0022034518800014 30290130 PMC6304695

[3] Jayaraman J Nagendrababu V Pulikkotil SJ Veettil SK Dhar V. Effectiveness of formocresol and ferric sulfate as pulpotomy material in primary molars: a systematic review and meta-analysis with trial sequential analysis of randomized clinical trials. Quintessence Int. (2020) 51(1):38–48. 10.3290/j.qi.a43617 31781690

[4] Shea BJ Grimshaw JM Wells GA Boers M Andersson N Hamel C Development of AMSTAR: a measurement tool to assess the methodological quality of systematic reviews. BMC Med Res Methodol. (2007) 7:10. 10.1186/1471-2288-7-10 17302989 PMC1810543

[5] Jayaraman J Nagendrababu V Pulikkotil SJ Innes NP. Critical appraisal of methodological quality of systematic reviews and meta-analysis in paediatric dentistry journals. Int J Paediatr Dent. (2018) 28(6):548–60. 10.1111/ipd.12414 30070003

[6] Jayaraman J Nagendrababu V. Quality of abstract of systematic reviews and meta-analyses in paediatric dentistry journals. Eur Arch Paediatr Dent. (2019) 20(5):383–91. 10.1007/s40368-019-00432-w 30887462

[7] Page MJ McKenzie JE Bossuyt PM Boutron I Hoffmann TC Mulrow CD The PRISMA 2020 statement: an updated guideline for reporting systematic reviews. Br Med J. (2021) 372:71. 10.1136/bmj.n71 PMC8005924 33782057

[8] Jayaraman J Dhar V Donly KJ Priya E Raggio DP Childers N Reporting StAndards for research in PedIatric dentistry (RAPID): an expert consensus-based statement. BMC Oral Health. (2021) 21:361. 10.1186/s12903-021-01698-7 34301229 PMC8299173

[9] Thang Le VN Kim JG Yang YM Lee DW. Risk factors for early childhood caries: an umbrella review. Pediatr Dent. (2021) 43(3):176–94. 34172110

[10] Gizani S Seremidi K Stratigaki E Tong HJ Duggal M Kloukos D. Vital pulp therapy in primary teeth with deep caries: an umbrella review. Pediatr Dent. (2021) 43(6):426–37. 34937612

[11] Fusar-Poli P Radua J. Ten simple rules for conducting umbrella reviews. Evid Based Ment Health. (2018) 21(3):95–100. 10.1136/ebmental-2018-300014 30006442 PMC10270421

[12] Dhar V Marghalani AA Crystal YO Kumar A Ritwik P Tulunoglu O Use of vital pulp therapies in primary teeth with deep caries lesions. Pediatr Dent. (2017) 39(5):146–59. 29070150

[13] Kirkham JJ Davis K Altman DG Blazeby JM Clarke M Tunis S Core outcome set-STAndards for development: the COS-STAD recommendations. PLoS Med. (2017) 14(11):e1002447. 10.1371/journal.pmed.1002447 29145404 PMC5689835

[14] Jayaraman J. Guidelines for reporting randomized controlled trials in paediatric dentistry based on the CONSORT statement. Int J Paediatr Dent. (2020) 31(Suppl 1):38–55. 10.1111/ipd.12733 32976673

